# Supplementary material for: Spiking neurons from tunable Gaussian heterojunction transistors
Source: Nat Commun. 2020 Mar 26;11:1565. doi: 10.1038/s41467-020-15378-7 (PMC7099079; doi:10.1038/s41467-020-15378-7)
Supplement: Supplementary file 1 — Supplementary Information [file 41467_2020_15378_MOESM1_ESM.pdf]

Spiking Neurons from Tunable Gaussian Heterojunction Transistors

Beck et al.

Supplementary Figures

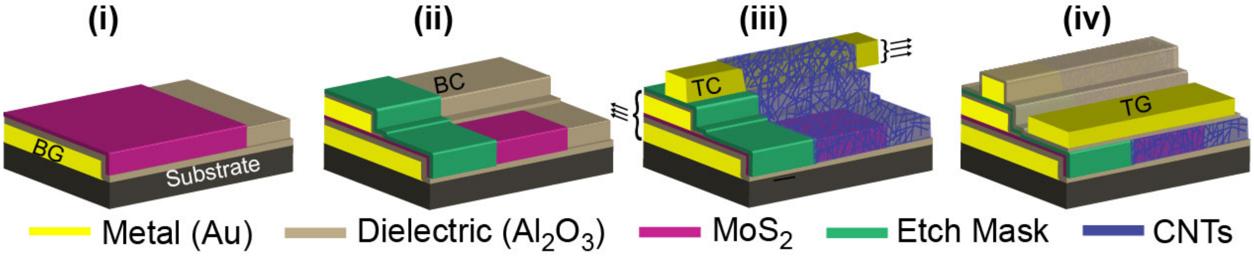

Supplementary Figure 1. Device structure throughout fabrication. (i) MoS_2 is transferred onto the self-aligned bottom gate (BG) and etched. (ii) Self-aligned bottom contact (BC) is deposited on MoS_2 followed by the few nm Al_2O_3 etch mask. (iii) Top contact (TC) is deposited on the BC followed by semiconducting single-walled carbon nanotube (CNT) network transfer and etching. (iv) Atomic layer deposition (ALD) Al_2O_3 is used to cover the entire device structure, after which the top gate (TG) is deposited and patterned.

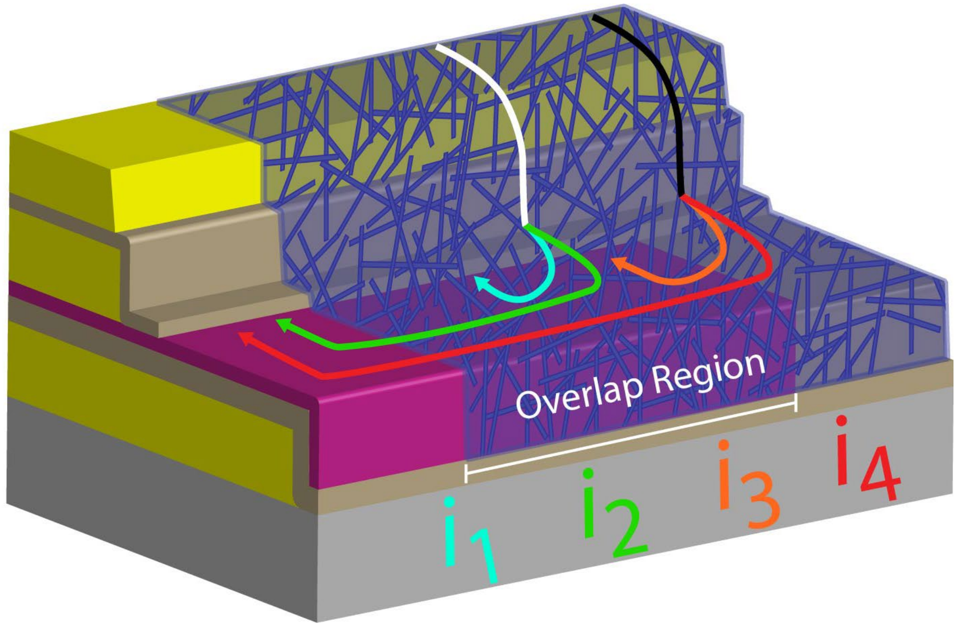

Supplementary Figure 2. Charge transport paths in GHeTs. The intentional offset between the MoS_2 (pink) and CNT (purple) semiconducting regions results in multiple charge transport paths with two possible current paths originating from the top contact at the overlap region (white) and the other two possible paths originating from the top contact away from the overlap region (black). The first current path (i_1, blue) is contained entirely in the overlap region, proceeding from the CNTs vertically down into the MoS_2. Note that this is the shortest path with a total device channel length < 1 µm (twice the dielectric extension of 300 nm plus the bottom (85 nm) and top (80 nm) contact heights). The second current path (i_2, green) starts with i_1 but proceeds vertically down into the MoS_2 and then laterally out of the overlap region. The third current path (i_3, orange) starts in the CNTs away from the overlap region and then proceeds laterally through the CNT film into the overlap region and vertically down into the MoS_2 film. The final current path (i_4, red) starts with i_3 but proceeds vertically down into the MoS_2 and then laterally out of the overlap region.

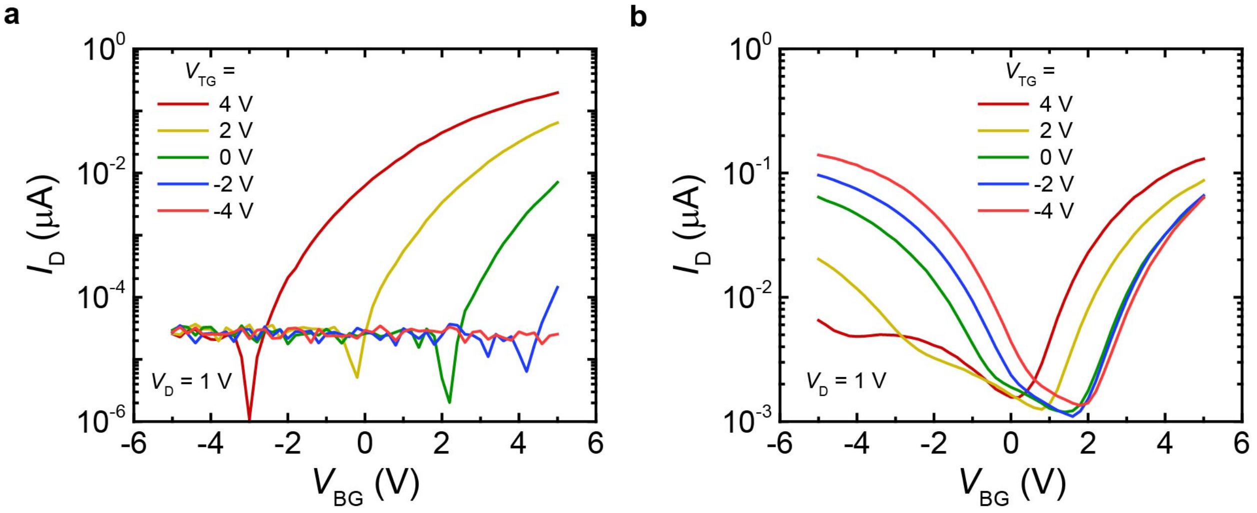

Supplementary Figure 3. Transfer characterization of control transistors. a, Transfer response of a dual-gated MoS_2 transistor showing n-type behavior and tunability of threshold voltage. **b**, Transfer response of a dual-gated CNT transistor showing ambipolar behavior and tunability of threshold voltages. The channel length of both devices is 50 μm. Because of the sub-micron channel length and semi-vertical structure of the GHeT, these measurements can only be used to qualitatively explain heterojunction device behavior. The exact threshold voltages and current levels will be impacted by the shorter channel length, heterojunction interface, and dielectric environment change because of the presence of the other semiconductor.

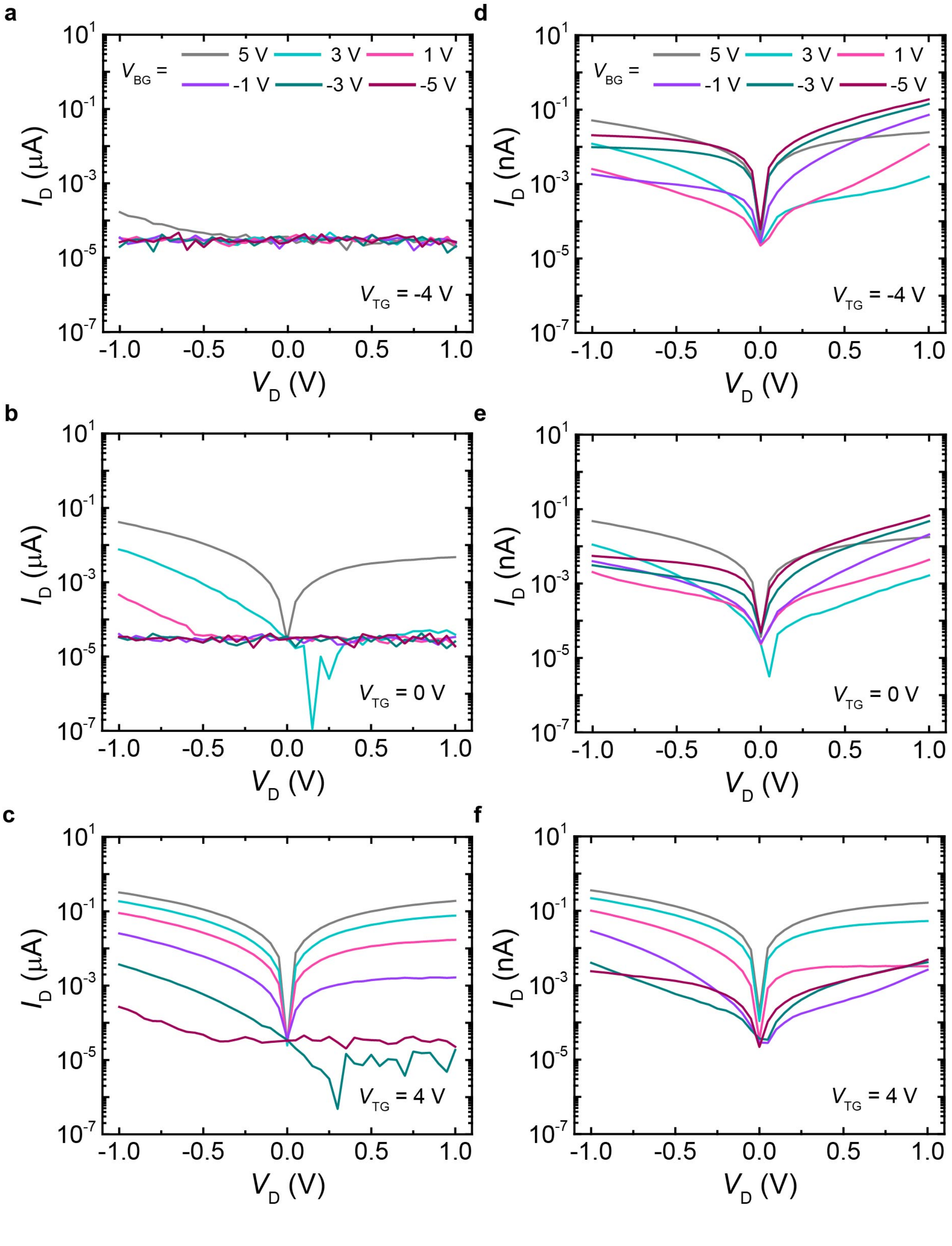

Supplementary Figure 4. Output characterization of control transistors. Output response of a dual-gated MoS_2 transistor for **a**, V_{TG} = -4 V, **b**, V_{TG} = 0 V, **c**, V_{TG} = 4 V and of a dual-gated CNT transistor for **d**, V_{TG} = -4 V, **e**, V_{TG} = 0 V, **f**, V_{TG} = 4. Because of the sub-micron channel length and semi-vertical structure of the GHeT, these measurements can only be used to qualitatively explain heterojunction device behavior. The exact threshold voltages and current levels will be impacted by the shorter channel length, heterojunction interface, and dielectric environment change because of the presence of the other semiconductor.

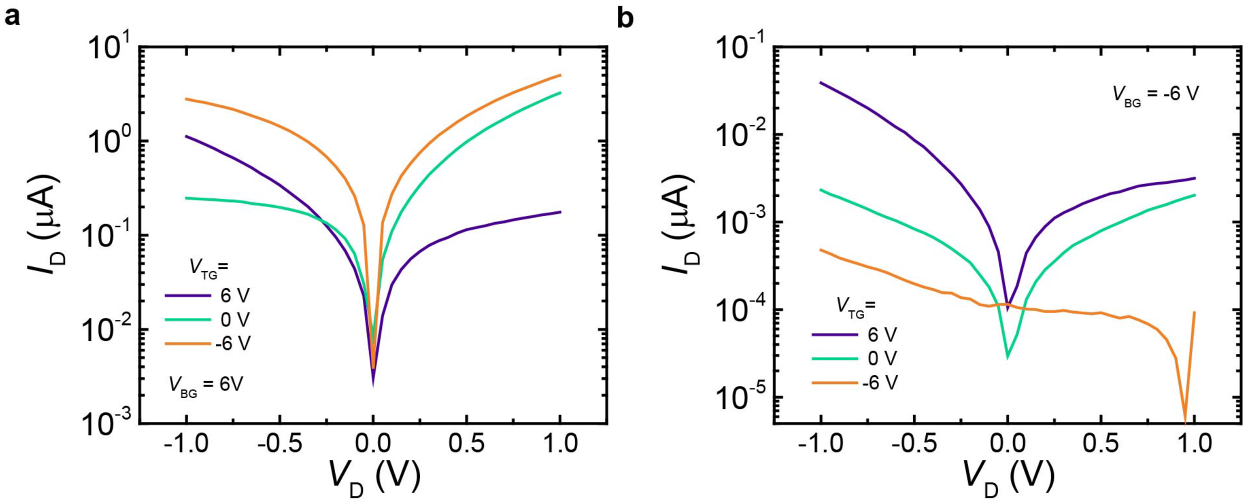

Supplementary Figure 5. Additional output responses for independent dual-gate operation. **a**, Output response of GHeT for V_{BG} = 6 V and V_{TG} = 6 V, 0 V, and -6 V. **b**, Output response of GHeT for V_{BG} = -6 V and V_{TG} = 6 V, 0 V, and -6 V. Note the different y-axis scale.

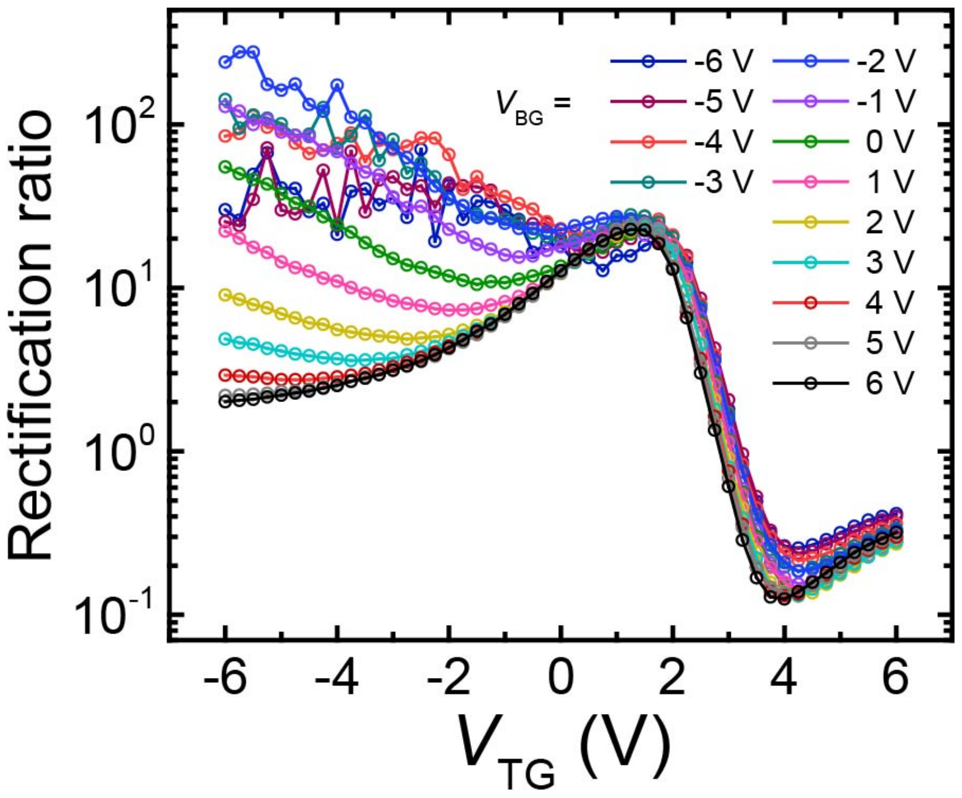

Supplementary Figure 6. Additional rectification ratios for independent dual-gate operation. Shown for V_{BG} = 6 V to V_{BG} = -6 V, the rectification ratio is defined as ratio I_D at V_D = 1 V divided by I_D at V_D = -1 V, as extracted from the transfer curve.

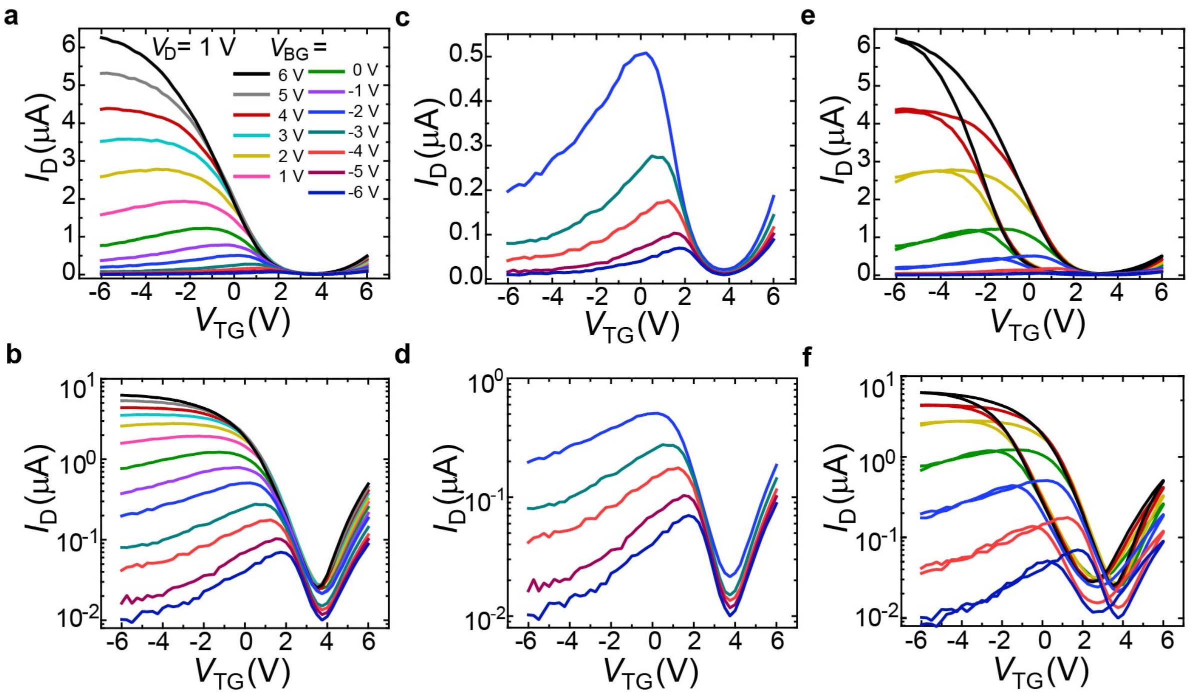

Supplementary Figure 7. Additional transfer responses for independent dual-gate operation. **a**, Transfer sweep for V_{BG} from 6 V to -6 V on a linear and **b**, log scale. **c**, Zoomed-in version of the transfer response for V_{BG} from -2 V to -6 V on a linear and **d**, log scale. **e**, Full sweep of the transfer response for selected V_{BG} on a linear and **f**, log scale showing expected hysteresis on the right side from CNTs. The legend in **a** corresponds to all plots.

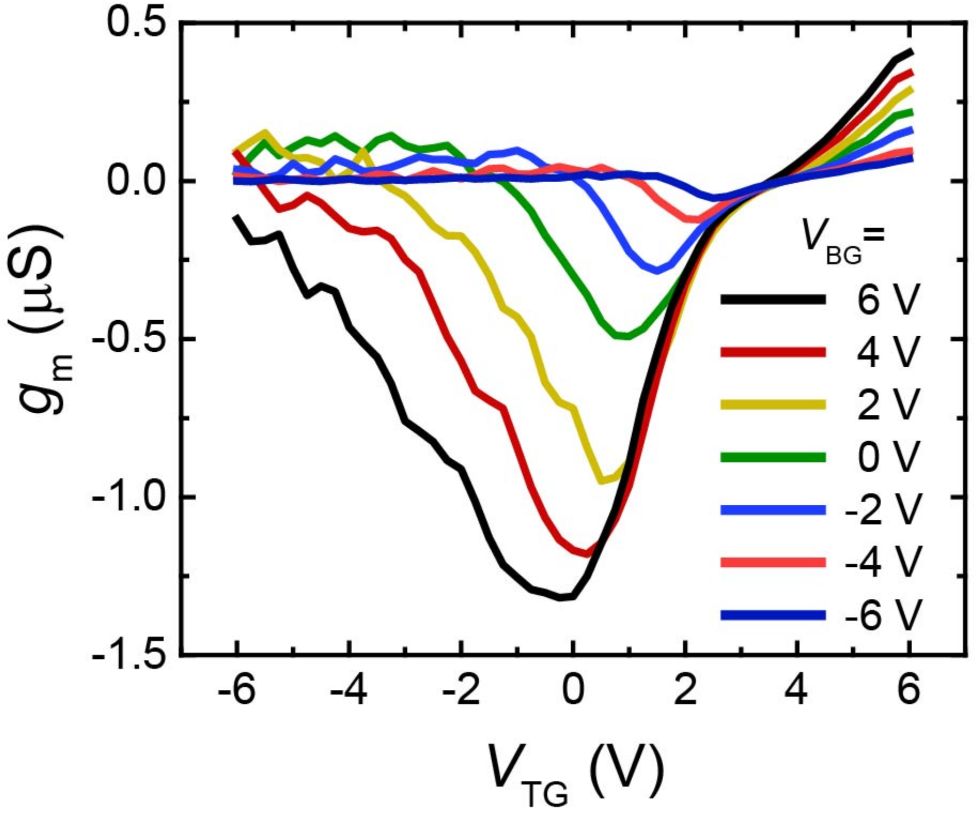

Supplementary Figure 8. Transconductance for independent gate operation. Values were extracted by calculating the slope of the raw data in Supplementary Figure 7.

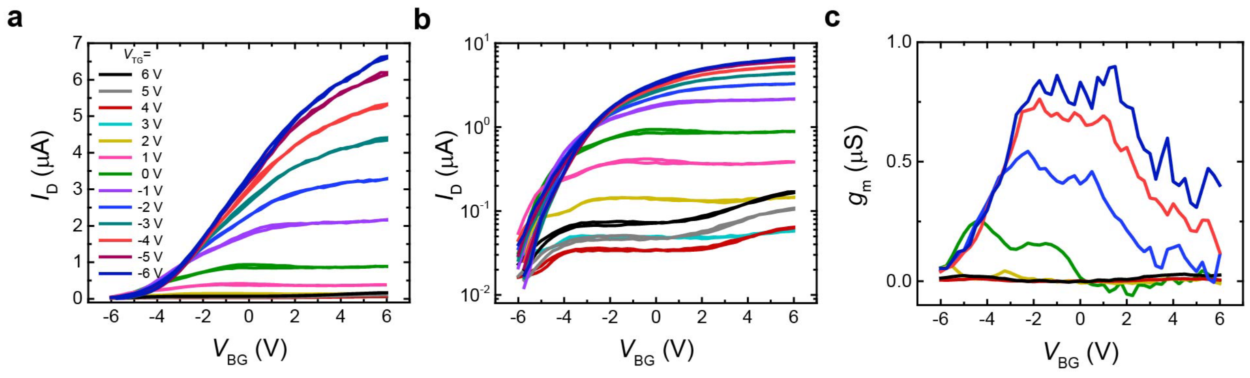

Supplementary Figure 9. Transfer response with respect to V_{BG} for independent dual-gate operation. **a**, Transfer sweep for V_{TG} from 6 V to -6 V on a linear and **b**, log scale. **c**, Transconductance extracted by calculating the slope of raw data in **a**. While V_{TG} has some electrostatic tunability of the MoS_2 response through the porous CNT film (Fig. 2c), V_{BG} has much less electrostatic control over the CNT response because of the continuous MoS_2 monolayer. The legend in **a** corresponds to all plots.

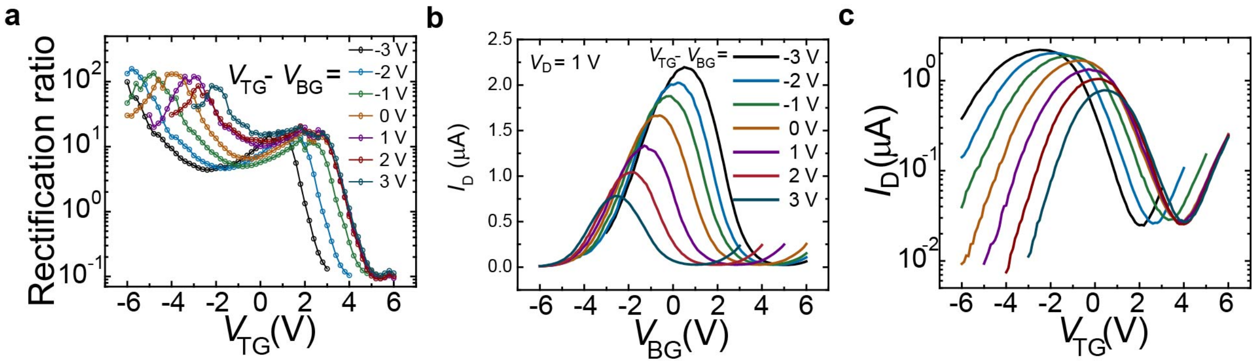

Supplementary Figure 10. Rectification ratio and transfer response for dependent dual-gate operation. **a**, Rectification ratio for $V_{TG} - V_{BG}$ from -3 V to 3 V with respect to V_{TG}, showing tunability over 3 orders of magnitude for all offsets. **b**, Transfer response with respect to V_{BG}, for simultaneous sweeping of both V_{BG} with V_{TG} on a linear scale. **c**, Transfer response with respect to V_{TG}, for simultaneous sweeping of both V_{BG} with V_{TG} on a log scale. The legend in **b** corresponds to **b** and **c**.

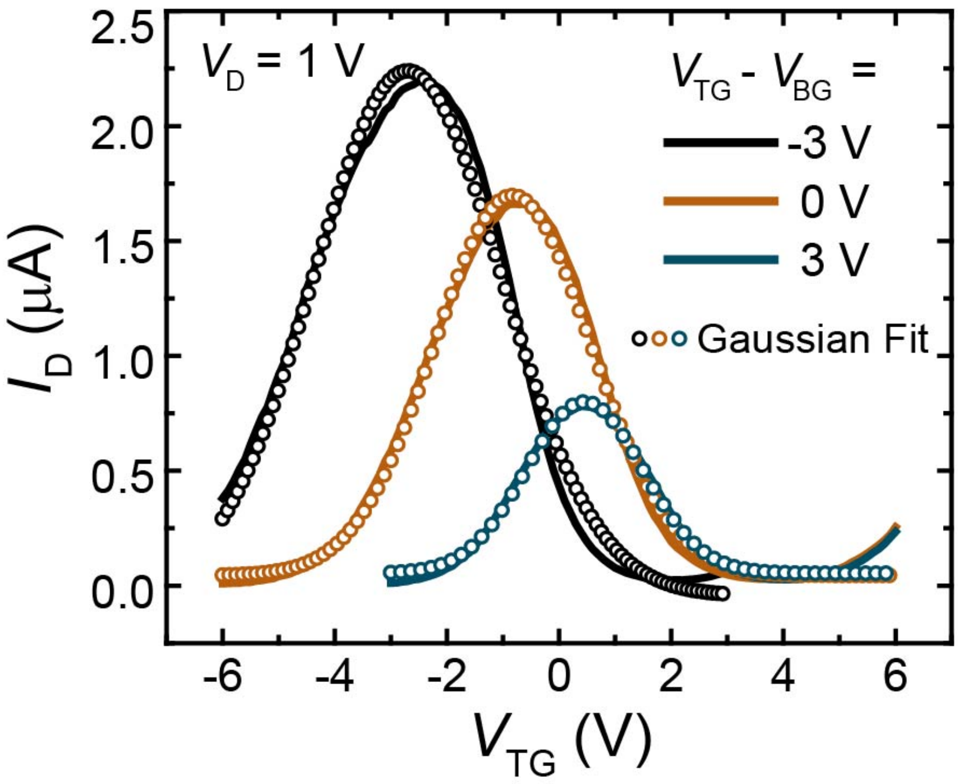

Supplementary Figure 11. Gaussian fits for dependent dual-gate operation. Gaussian fitting of representative curves from Fig. 3a highlights the consistently symmetric response for dependent biasing without a significant loss of peak current.

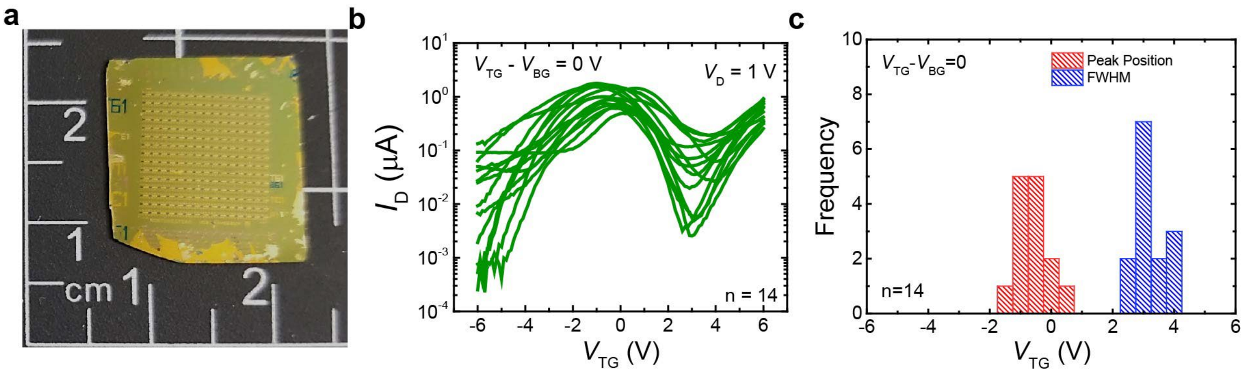

Supplementary Figure 12. Large-area device statistics. **a**, Optical image of 1 cm x 1 cm device array containing ~0.5 cm x 0.5 cm area of continuous MoS_2. **b**, Transfer response with respect to V_{TG} for $V_{TG} – V_{BG}$ = 0 V for 14 different GHeTs under identical biasing conditions. Variability can be attributed to non-uniformities in the CNT network or bilayer regions and grain boundaries in the MoS_2 film. **c**, Extracted peak positions and FWHM from Gaussian fits of curves shown in **b** with an average peak position of -0.42 V ± 0.55 V and average FWHM of 2.92 V ± 0.48 V. Source data are provided as a Source Data file.

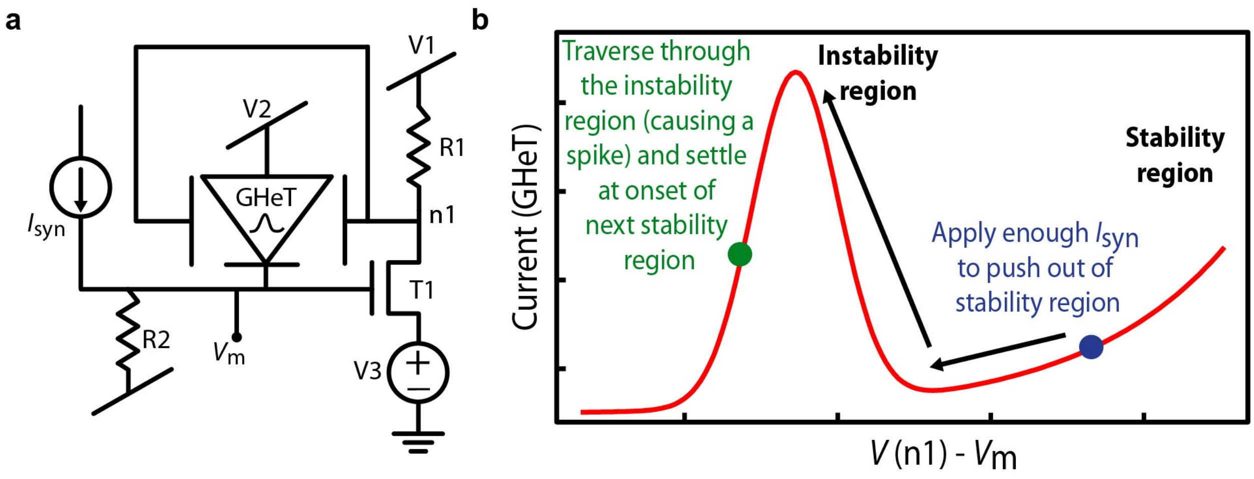

Supplementary Figure 13. Basis for using the GHeT response to mimic g_{Na}. **a**, Circuit schematic that exploits the unique relationship between the GHeT transconductance (g_m) and V_{TG} to mimic the time-dependent peaked response of g_{Na}. **b**, Expected behavior of a GHeT as V_{n1}-V_m changes. The drain of the GHeT is connected to potential V2, the source is connected to V_m (i.e., the node integrating the synapse current I_{syn}), and the two gate-terminals are shorted and connected to the output node n1 of the T1-R1 amplifier. The loop gain from V_{n1}-V_m is proportional to -$g_{T1}\times g_m$. When V_m is zero and V_{n1} is high, the GHeT is in a positive g_m region, so the loop gain of V_{n1}-V_m is negative and circuit rests at a stable operating point (blue). Input of a constant synaptic current, I_{syn}, increases V_m, (balanced by the leaking resistor, R2). If I_{syn} is adequately high, V_m increases sufficiently to turn on T1, causing V_{n1} to drop. With decreasing V_{n1}-V_m, g_m becomes negative, and the loop-gain for V_{n1}-V_m becomes positive. This condition generates a spike in V_m. Finally, with increasing V_m, the GHeT again reaches a positive g_m, the loop gain becomes negative, and the circuit resets and settles at the onset of the next stability region (green).

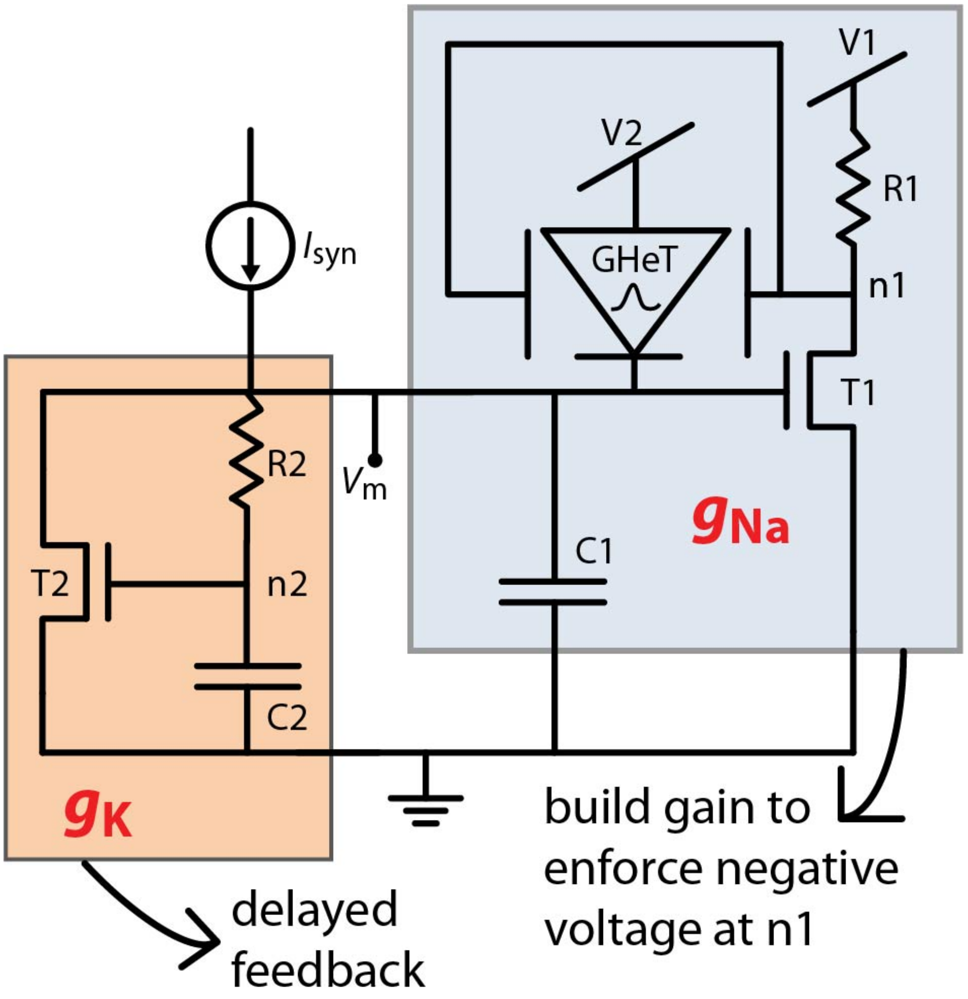

Supplementary Figure 14. GHeT-based spiking neuron circuit. The GHeT device along with T1-R1-C1 emulates the Na^+ ion channel conductance (g_{Na}) while T2-R2-C2 emulates the K^+ ion channel conductance (g_K). In this circuit, the conductivity of GHeT is exploited for negative gate-to-source bias to induce spiking. The gates of the GHeT device are controlled using common source (CS) amplifier (formed by T1-R1) negative gain stage. For sufficiently high synapse current (I_{syn}), load C1 integrates the I_{syn} and OFF current from the GHeT (I_{OFF}) (i.e., source bias of GHeT (V_m) increases with time with a slope proportional to I_{syn} + I_{OFF}). The potential V_m in turn biases the CS stage as well as the delayed feedback stage. As V_m exceeds the threshold voltage of T1, voltage applied to the gates of the GHeT is pulled down due to the negative gain of the CS stage ($V_{n1} = -g_{m,T1} \text{ x } V_m$), resulting in negative gate-to-source voltage across the GHeT ($V_{GS} = -g_{m,T1} \text{ x } V_{n1}-V_m$). This forces GHeT to conduct maximum current (I_{PEAK}), increasing the slope of V_m in proportion to ($I_{syn} + I_{PEAK}$). As V_m continues to increase, V_{n1}-V_m continues to decrease, accessing the positive g_m of the GHeT and V_{n2} exceeds the T2 threshold voltage, allowing g_K to initiate and reset V_m.

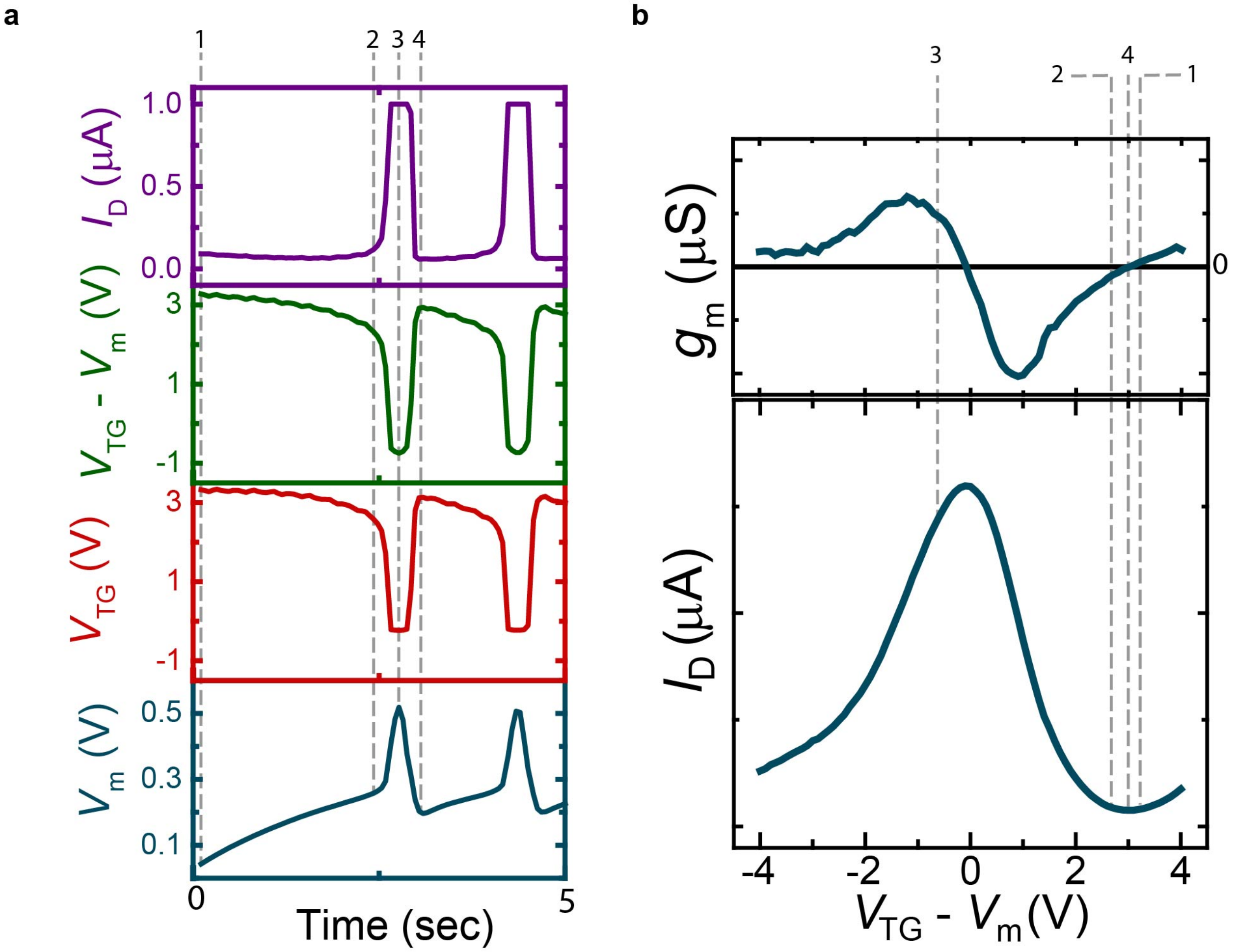

Supplementary Figure 15. Spike evolution with respect to GHeT. **a**, Temporal evolution of GHeT source voltage, V_m, applied top gate voltage, V_{TG}, relative gate bias, V_{TG}-V_m, and drain current, I_D, during the first 5 seconds of the constant spiking behavior shown in Fig. 4. **b**, Transconductance and current evolution as a function of gate bias for the GHeT device used in Fig. 4. The states of various elements in the circuit for the numbered positions are detailed in Supplementary Table 1.

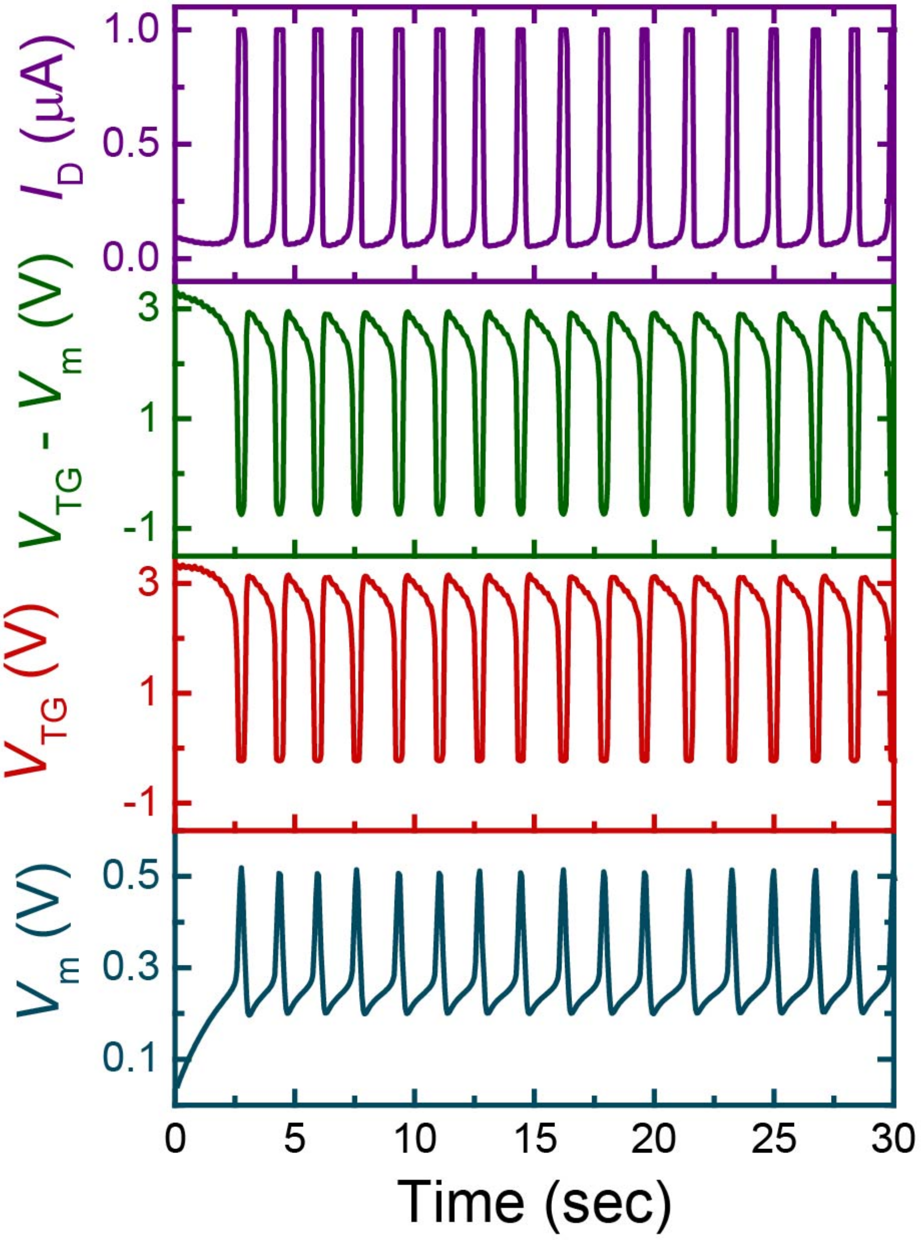

Supplementary Figure 16. GHeT gate voltage and current during spiking. Experimental results for the actual voltage applied to the top gate and the corresponding current flowing through the GHeT while functioning in the spiking neuron circuit detailed in Fig. 4d. The consistency of spiking in V_m with the gate voltage and current of the GHeT affirms that the spiking response originates from the GHeT device.

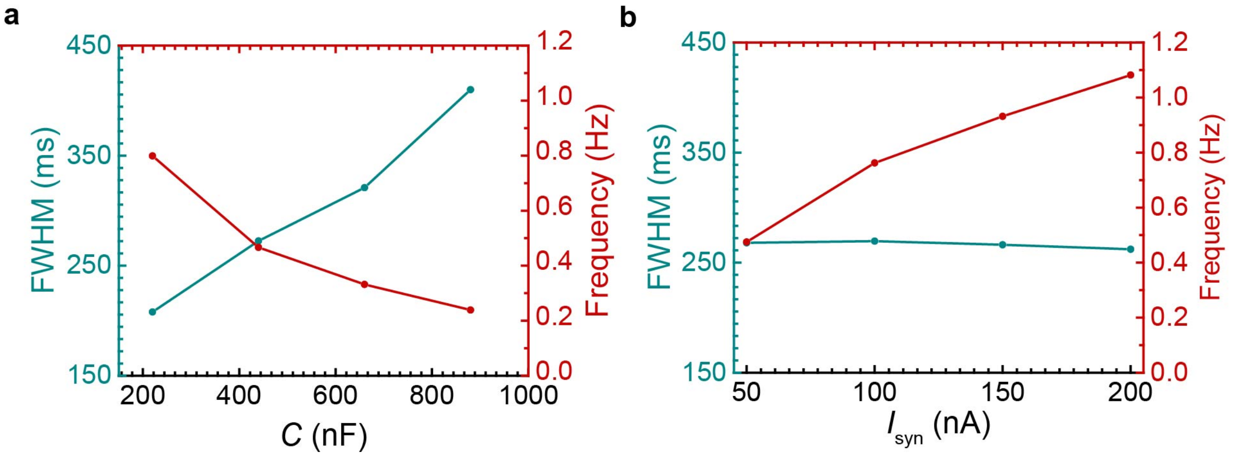

Supplementary Figure 17. Changes in FWHM and spiking frequency with varied capacitances and I_{syn} based on simulations. **a**, The FWHM of a given spike increases and the frequency of spiking decreases with increasing capacitance values (C1 and C2). **b**, The FWHM stays fairly constant and the frequency of spiking increases proportionally with increasing I_{syn}.

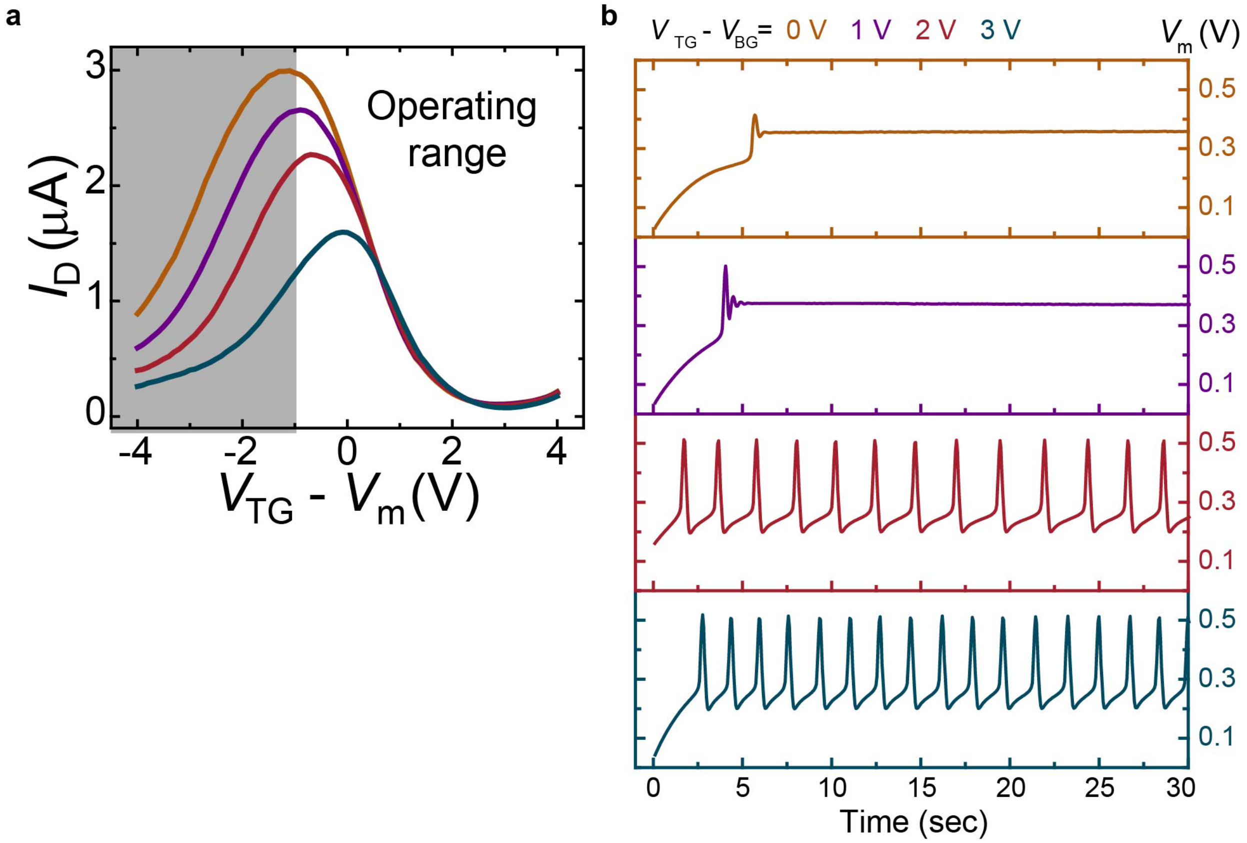

Supplementary Figure 18. Experimental neuron spiking controlled by gate offset. **a**, Transfer characteristics from the specific device used in experimental circuit demonstration. **b**, Experimental results for the first 30 seconds from circuit detailed in Fig. 4d. The bias offset, V4, between the V_{TG} and V_{BG} determines the spiking response of the circuit. When the circuit operating region (4 V to -1 V, shaded in **a**), does not contain both negative and positive g_m values near I_{PEAK} of the Gaussian transfer response (brown and purple), g_K is unable to reset V_m below the threshold of T1 resulting in no spiking. If the operating region contains both negative and positive g_m values near I_{PEAK} of the Gaussian transfer response (red and blue), g_K resets V_m below the threshold of T1, thereby resetting the GHeT. This results in constant spiking for as long as I_{syn} is applied.

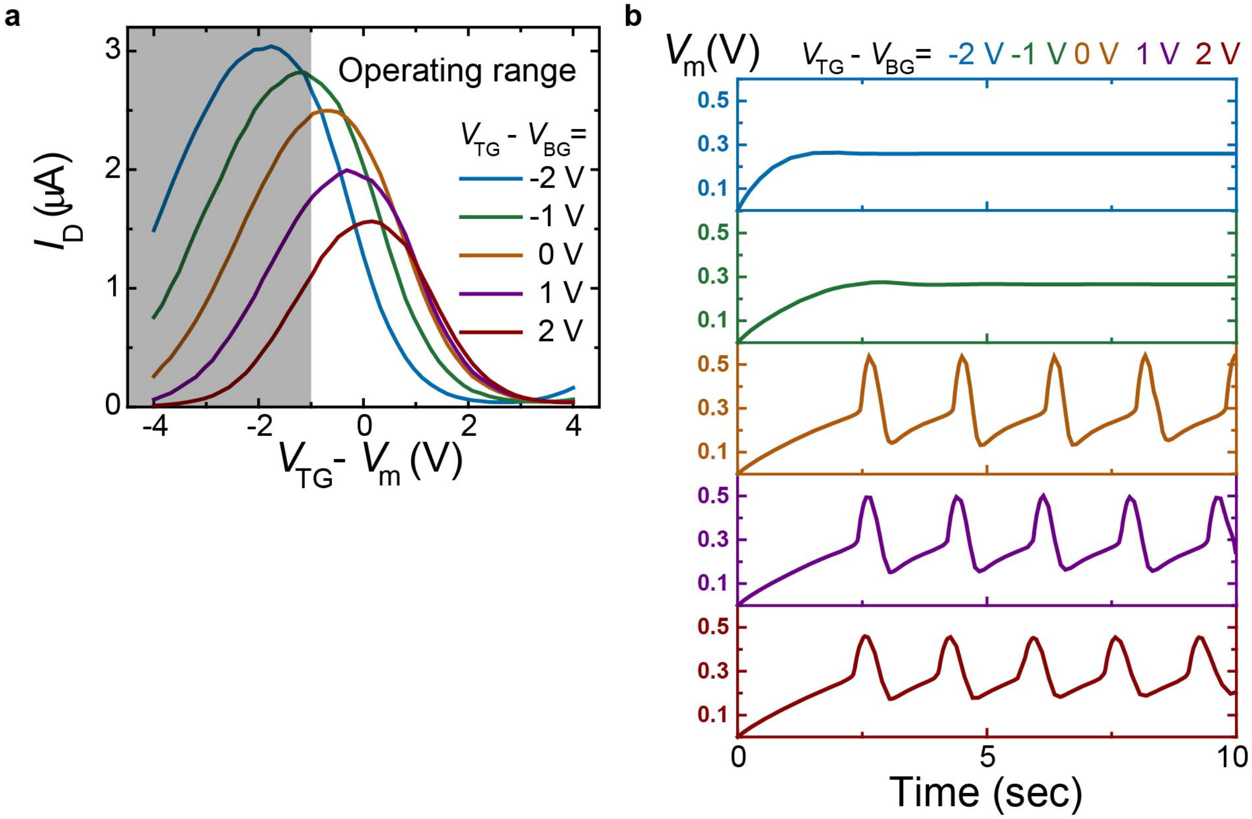

Supplementary Figure 19. Simulated neuron spiking controlled by gate offset. **a**, Experimental data from a prototypical GHeT device used in the simulations. **b**, Simulations of the spiking neuron circuit to corroborate the experimental results showing that the offset between the gates can be used to suppress the spiking response of the circuit. The circuit parameters vary slightly from those used for the experimental demonstration to account for various non-idealities.

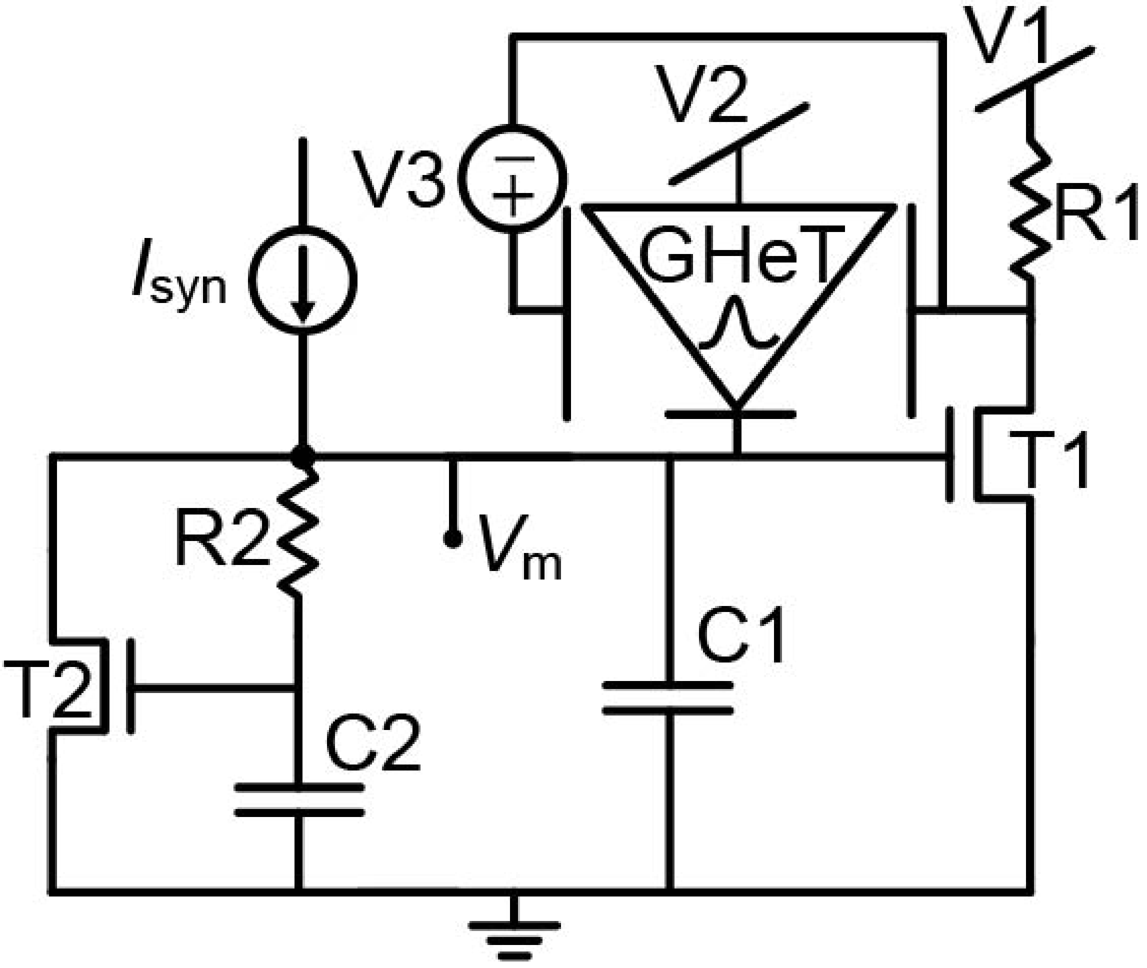

Supplementary Figure 20. Circuit for constant and class I spiking. Constant spiking is achieved using a 40 nA synapse current pulse for 25 sec (Fig. 5a). If the synapse current is increased linearly from 0 nA to 80 nA over 75 sec (Fig. 5b), class I spiking occurs. This circuit design matches the experimental demonstration of constant spiking.

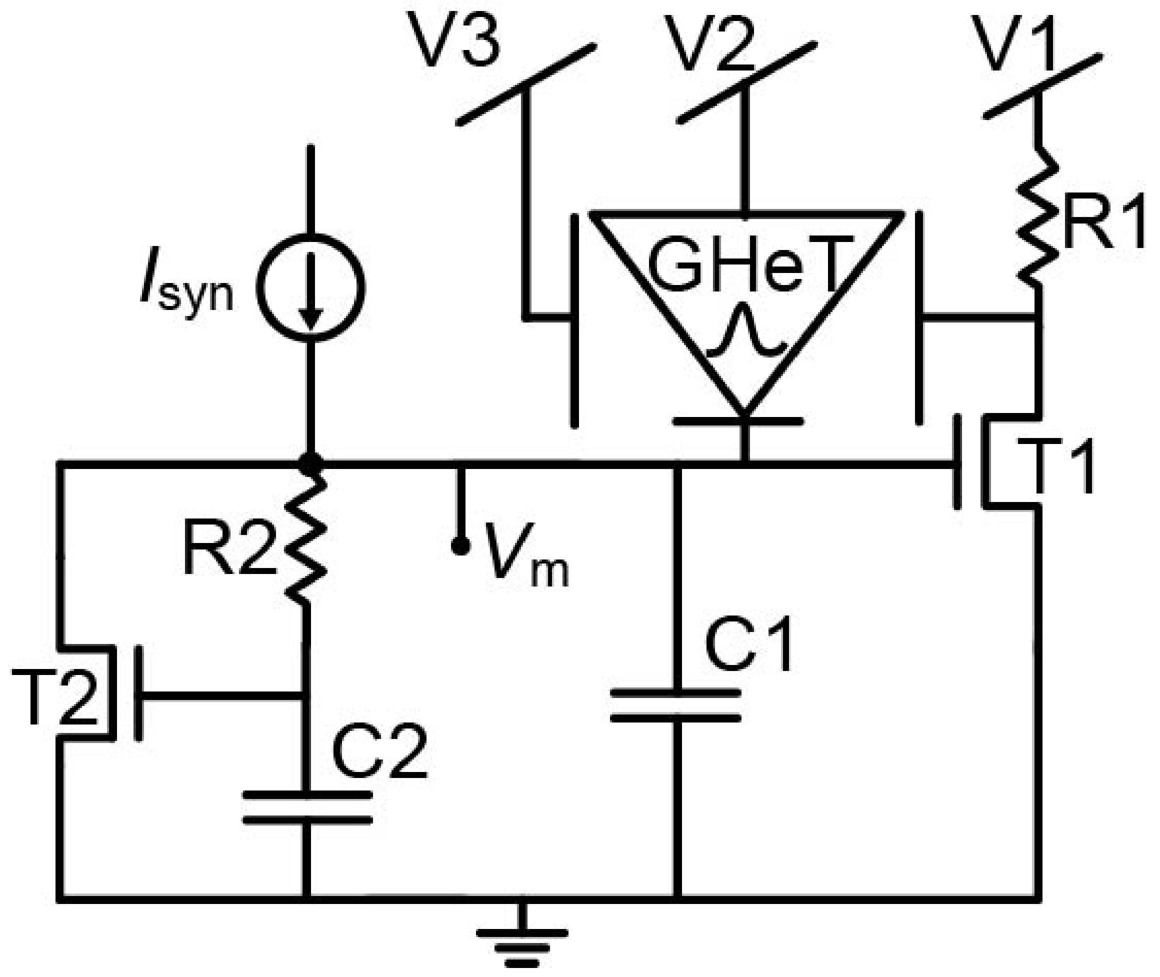

Supplementary Figure 21. Circuit for spike latency, integrator, and phasic spiking. The circuit has been modified from Supplementary Figure 14 to allow independent biasing of the bottom gate of the GHeT. A bottom gate bias (V3) of 2.5 V enables spike latency for 0.4 sec synapse pulse of 50 nA (Fig. 5c) and integration of spikes when 0.4 sec synapse pulses of 35 nA are within 0.3 sec of each other (Fig. 5d). Phasic spiking occurs by biasing V3 at 3.1 V using a 40 nA synapse pulse for 25 sec (Fig. 5e).

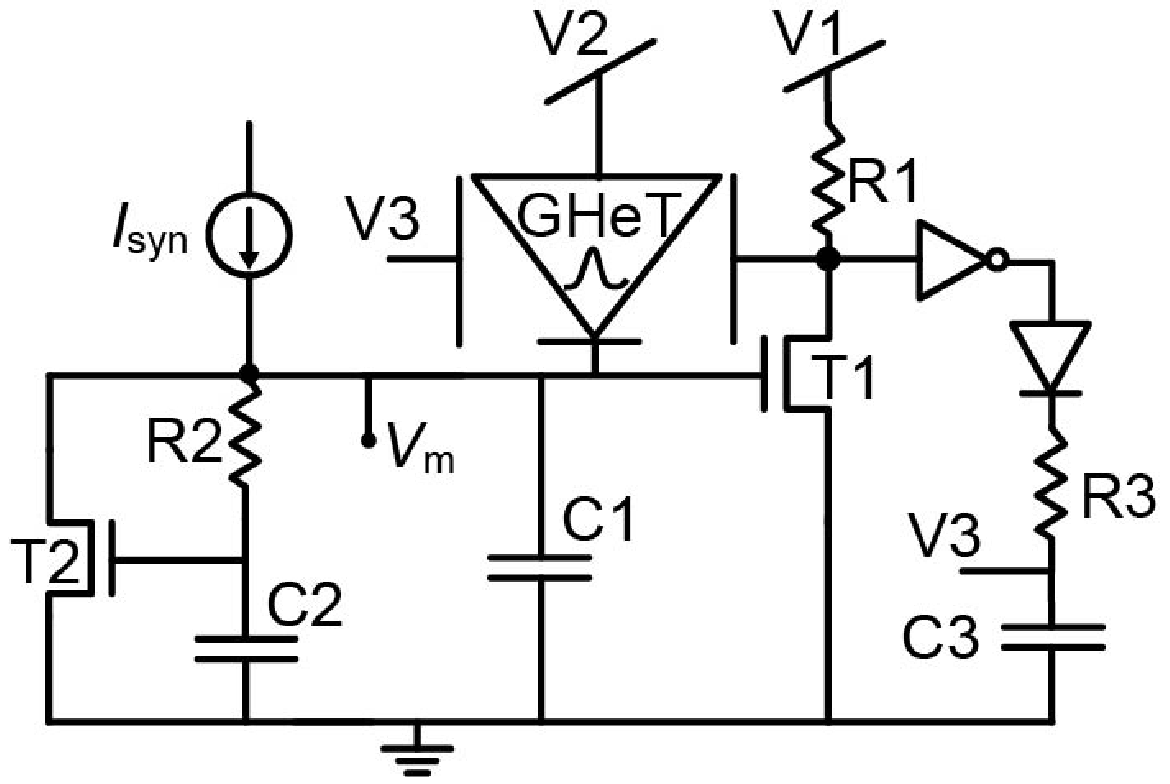

Supplementary Figure 22. Circuit for phasic bursting. For phasic bursting, the loss of the positive g_m for independent gate biasing above V_{BG} = 3.5 V (Supplementary Figure 4) is exploited by controlling the bias at V3 through controlled, spiking dependent charging of C3 (Fig. 5f).

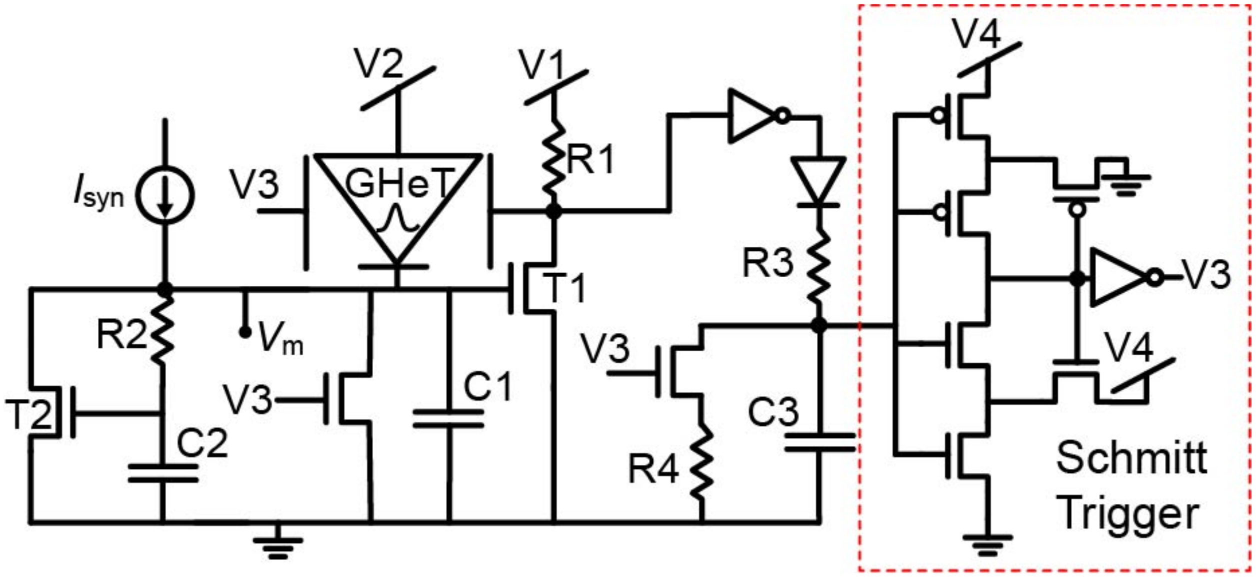

Supplementary Figure 23. Circuit for tonic bursting. In addition to spiking-dependent charging of C3, a Schmitt Trigger circuit and two additional transistors are incorporated to control the biasing at V3, thus enabling tonic bursting (Fig. 5g).

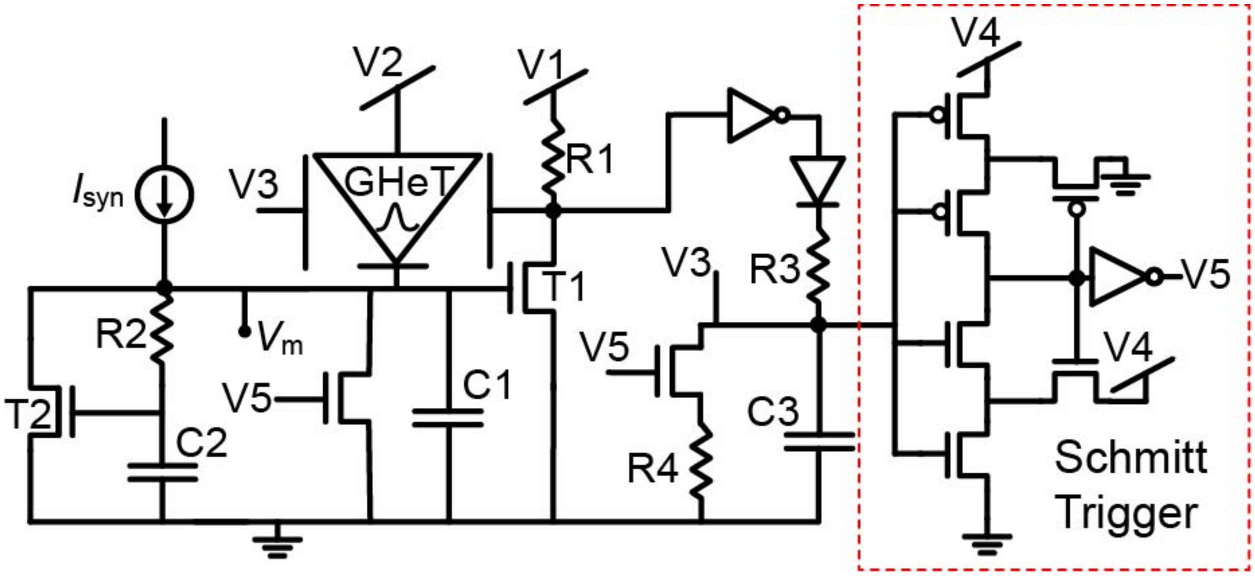

Supplementary Figure 24. Circuit for dampened tonic bursting. Compared to Supplementary Figure 17, here, V3 is directly connected to C3, causing the bottom-gate bias to gradually increase as the spiking burst occurs, thereby reducing the positive g_m of the GHeT. This condition results in a dampening of the spiking response (Fig. 5h).

Supplementary Tables

Supplementary Table 1. State of various elements in the circuit at numbered positions in Supplementary Figure 15.

**Position**	V_m	**T1**	**C1 and C2**	**T2**	**GHeT Gate (V_{TG})**	**State of GHeT**	g_m
1	0 V	OFF	Starts to charge	OFF	~ 3.3 V	OFF	> 0
1 to 2	Increasing with $I_{syn} + I_{OFF}$	OFF	Charges	OFF	Decreases with V_m	OFF	> 0 to < 0
2	~ 0.25 V ~ $V_{th,T1}$	Turns ON	Charges	OFF	~ 2.5 V	OFF	< 0
2 to 3	Increasing with $I_{syn} + I_{PEAK}$	ON	Charges	OFF	Drops as T1 is ON	Turns ON with increasing current through I_{PEAK} then decreasing current with increasing V_m	< 0 to > 0
3	~ 0.5 V ~ $V_{th,T2}$	ON	Starts to Discharge	Turns ON	~ -0.75 V	ON $I_D < I_{PEAK}$	> 0
3 to 4	Decreasing due to pull-down path	Turns OFF	Discharges	Turns OFF	Spikes as T1 turns OFF	Turns OFF through I_{PEAK} with decreasing current as V_m drops and V_{TG} spikes.	> 0 to ~ 0
4	~ 0.2 V < $V_{th,T1}$	OFF	Starts to charge	OFF	~ 3 V	OFF	~ 0

Supplementary Table 2. Simulation parameters for spiking neuron circuits. The table details the parameters used for the simulation corresponding to each manuscript figure. For all simulations, the other parameters did not change (V1 = 4 V, V2 = 1 V, C2= 0.22 µF, R1 = 100 kΩ and R2= 1.5 MΩ).

**Manuscript Figure**	**V3**	**V4**	**V5**	**C1**	**C3**	**R3**	**R4**	I_{syn} (timing)
4f	-210 mV	n/a	-10 mV	0.44 µF	n/a	n/a	n/a	50 nA (constant)
5a (Constant)	0 V	n/a	n/a	0.22 µF	n/a	n/a	n/a	40 nA (10-35 s)
5b (Class 1)	0 V	n/a	n/a	0.22 µF	n/a	n/a	n/a	0-80 nA (linearly over 10-85 s)
5c (Latency)	2.5 V	n/a	n/a	0.22 µF	n/a	n/a	n/a	50 nA (10-10.4 s)
5d (Integrator)	2.5 V	n/a	n/a	0.22 µF	n/a	n/a	n/a	35 nA (0.4 s pulses at 10, 10.7, 40, and 42 s)
5e (Phasic)	3.1 V	n/a	n/a	0.22 µF	n/a	n/a	n/a	40 nA (10-35 s)
5f (Phasic bursting)	n/a	n/a	n/a	0.22 µF	0.3 µF	1.5 MΩ	n/a	40 nA (10-35 s)
5g (Tonic bursting)	n/a	5 V	n/a	0.22 µF	0.3 µF	2 MΩ	6 MΩ	40 nA (10-35 s)
5h (Dampened tonic bursting)	n/a	5 V	n/a	0.22 µF	0.3 µF	1.5 MΩ	6 MΩ	40 nA (10-35 s)
